# Required force for distraction during medial opening wedge high tibial osteotomy may serve as a predictive indicator for lateral hinge fracture

**DOI:** 10.1002/jeo2.12086

**Published:** 2024-07-06

**Authors:** Baran Soykan, Arman Vahabi, Elcil Kaya Biçer, Bora Uzun, Semih Aydoğdu

**Affiliations:** ^1^ Department of Orthopaedics and Traumatology Ege University School of Medicine Izmir Turkey; ^2^ Department of Biomechanics Dokuz Eylül University Izmir Turkey

**Keywords:** correction force, distraction, high tibial osteotomy, lateral hinge, medial open wedge

## Abstract

**Introduction:**

Medial open wedge high tibial osteotomy is a biological procedure for treating unicompartmental knee osteoarthritis. The literature repeatedly highlights the significance of preserving an intact lateral hinge during this procedure. We investigated the temporal course of distraction forces during distraction at the osteotomy site, aiming to quantitatively measure and analyse temporal changes in distraction forces at different distraction points for intact and fractured lateral hinges.

**Materials and Methods:**

This biomechanical study was conducted on 10 human cadavers, which were divided into two groups: one with preserved 1 cm intact lateral cortexes (ILCs) and the other with completely osteotomised fractured lateral cortexes (FLCs). An experimental setup was custom designed to facilitate the required force measurement during distraction. The distraction forces were recorded with a force gauge at 0.5‐mm intervals throughout the distraction.

**Results:**

There was a significant difference between the ILC and FLC groups in distraction forces at all points (8–15 mm). The ILC group consistently exhibited higher distraction force values, with FLC recording values ranging from 8.8% to 13.2% of ILC's. Lateral hinge fractures caused an 86.7% reduction in the initial required force for distraction, significantly impacting the force required for distraction. The ILC group displayed a linear increase in the required distraction force up to 12.5 mm of distraction, which reached 3.7 times the initial value at 12.5 mm of distraction. The FLC group had lower baseline required distraction forces, following a relatively linear trend with more limited increases.

**Conclusion:**

FLCs in medial opening wedge osteotomy are associated with significant reductions in the force required for distraction, and a sudden decrease in distraction force during distraction may indicate a lateral hinge fracture. Force measurement devices for use during distraction could offer valuable insights and provide surgeons with immediate warnings for LHFs.

**Level of Evidence:**

Level IV.

**Abbreviations**
FLC: fractured lateral cortex
HTO: high tibial osteotomy
ILC: intact lateral cortex
LHF: lateral hinge fracture
MCL: medial collateral ligament
MOWHTO: medial opening wedge high tibial osteotomy
RFD: required force for distraction

## INTRODUCTION

Medial opening wedge high tibial osteotomy (MOWHTO) is a biological intervention that is favoured for young patients with medial compartment osteoarthritis. In the context of medial compartment osteoarthritis, high tibial osteotomy can be performed using various techniques, including medial open wedge tibial osteotomy, lateral closed wedge tibial osteotomy and dome osteotomy [11]. Achieving optimal medial distraction is essential during MOWHTO surgery, and this is best accomplished by preserving the lateral cortical hinge. The literature has consistently emphasised the importance of protecting a lateral cortical hinge of at least 1 cm during osteotomy [3, 6, 20]. Failing to preserve the lateral cortical hinge can result in instability at the osteotomy site, which may have significant clinical consequences, including loss of correction, impaired postoperative rehabilitation, delayed union, nonunion and implant failure. Lateral hinge fractures (LHFs) are among the most common complications of MOWHTO, with reported rates of 3%–50% [2, 15, 22].

In addition to the technical challenges of preserving lateral cortical hinges during MOWHTO procedures, another challenge is recognising LHFs intraoperatively or even postoperatively [6, 16]. A recent systematic review reported 40% LHFs in one study, 10% of which were missed using plain radiographs and fluoroscopy [15]. LHFs, which are often not evident on standard radiographs, can only be discerned using computed tomography scans. Consequently, intraoperative detection of LHFs may be limited by inadequate imaging in the operating room [16]. LHFs that go unrecognised during the surgical procedure and are not addressed appropriately can increase the risk of postoperative complications. The fact that effectively managed LHF cases can have similar clinical outcomes to cases without LHFs emphasises the importance of diagnosing and addressing LHFs [7, 14].

Although some researchers have evaluated soft tissue balance in knee arthroplasty using distraction force measurement [10, 18, 21], no study, to our knowledge, has considered the implications of distraction forces applied along the osteotomy line, which could potentially predict and identify LHFs.The aim of this study was to analyse the temporal course of distraction forces during MOWHTO and to assess the impact of LHFs on distraction forces. We sought to determine whether LHFs could be quantitatively recognised by precisely measuring alterations in distraction forces. Furthermore, we studied the feasibility of quantifying the temporal course of distraction forces throughout the distraction procedure. The hypothesis of the study was that the distraction force trajectory during distraction could exhibit a specific pattern that could be used to predict and identify LHFs.

## MATERIALS AND METHODS

The entire legs of the cadavers were acquired from the Science Care Company. No specific age limit was applied to the cadavers, which had no recorded history of prior surgical procedures related to the lower extremities, showed no radiological signs of moderate or severe osteoarthrosis (Kellgren–Lawrence Grade 3/4) [12] or signs of previous trauma, exhibited no major deformities and possessed normal tibiofemoral alignment.

The cadavers were initially deep frozen at −18°C until experimentation. Prior to the experiment, the cadavers were extracted from the freezer and transferred to a refrigerator set at +4°C to control the thawing process. After 24 h of thawing, the experimental procedures were conducted. The cadavers were categorised into two distinct groups based on the presence or absence of lateral cortex fractures. In the first group, consisting of five cadavers, 1 cm of intact bone was maintained within the lateral cortex during osteotomy, which was conducted under fluoroscopy guidance. A 1 cm lateral cortical hinge was preserved using osteotomes with depth markings, consistent with measurements made via k‐wires placed under fluoroscopic control. This group was labelled the intact lateral cortex (ILC) group. In the second group, comprising five cadavers, osteotomy was extended to the lateral cortex, in line with the initial osteotomy, again under fluoroscopic control. This group was labelled the fractured lateral cortex (FLC) group [23].

**Figure 1:**
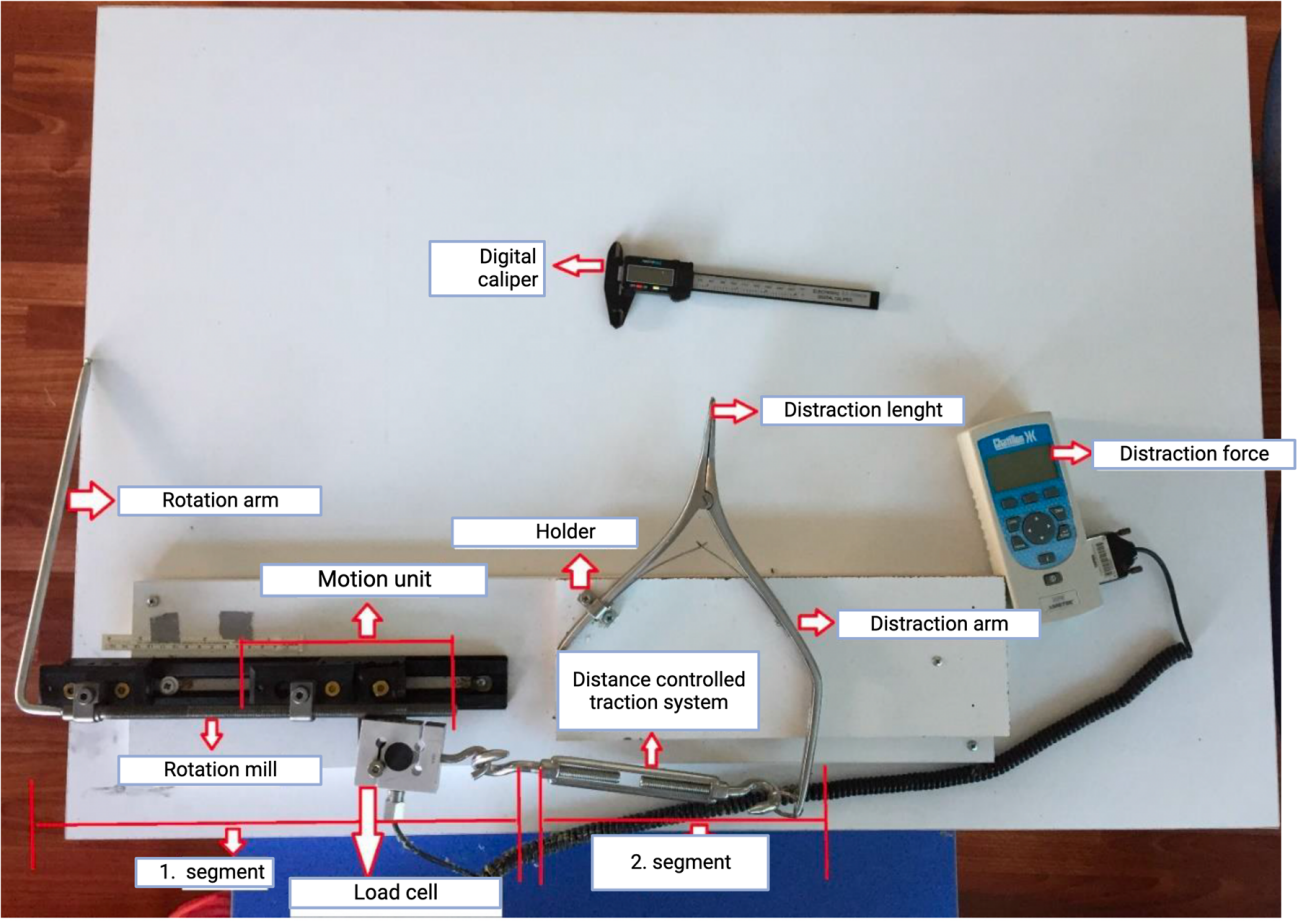
Custom made experimental setup.

An experimental system was custom designed, which consisted of two components. The first included a rail system and a motion unit that moved horizontally with the manual rotation of the rotation shaft. A force gauge device (Ametek DFS), connected to the load cell, measured real‐time distraction forces. The operational principle underpinning this system was related to the manual rotation of the rotation arm, which made the motion unit linked to the rotation shaft traverse the rail system. The distractor arm, connected to the motion unit via a traction system, moved synchronously with the motion unit and opened the distractor tips (placed along the osteotomy line). The opening distance between the distractor tips situated along the tibia osteotomy line was assessed using a digital caliper, and the distraction forces were quantified using the load cell on the motion unit (see Figure 1).

During the surgical procedure, the joint capsule and ligaments were left undisturbed. Subsequently, the gracilis tendon was elevated alongside the sartorius fascia, and the pes anserinus was retracted to expose the superficial fibres of the medial collateral ligament, the distal attachment of which was preserved. Then, two k‐wires were inserted towards the upper point of the fibular head. These wires were placed approximately 4 cm below the level of the medial femorotibial joint. Using a cutting motor, a medial–lateral cut was made in the proximal tibia, following the k‐wires closely while preserving the integrity of the lateral cortex in the ILC group (leaving 1 cm of cortical hinge). In the FLC group, osteotomy was completed without leaving any lateral cortical hinge. Osteotomy was completed uniplanarly with osteotomes.

**Figure 2:**
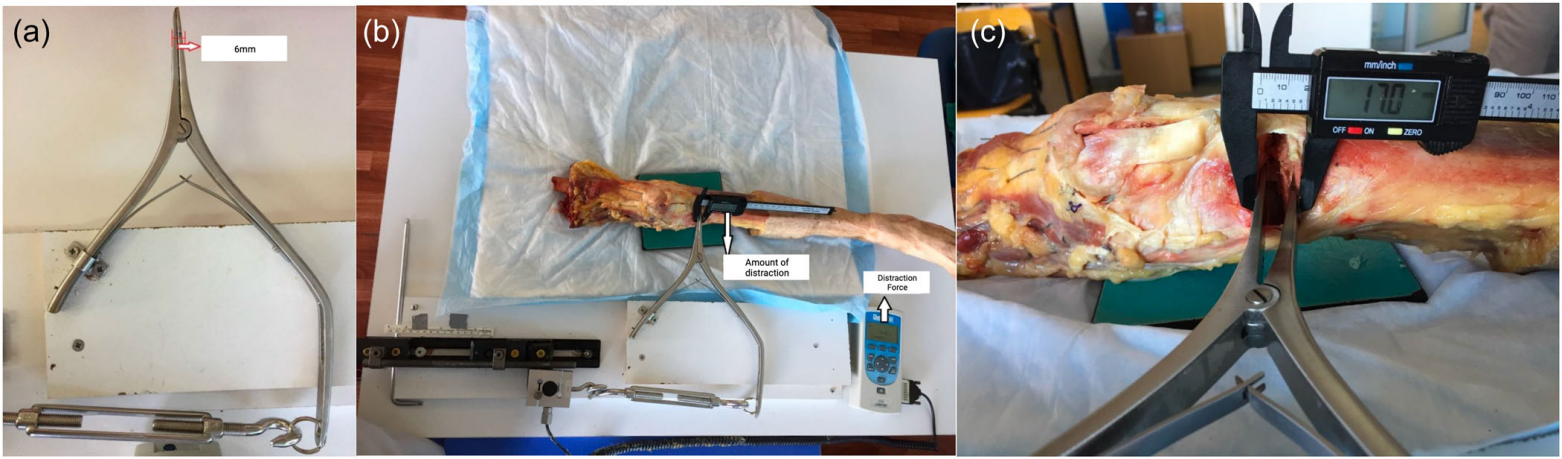
(a) Marked tip of distractor at 6 mm width; (b) cadaver placed in custom‐made setup; (c) distraction measurement simultaneously with force monitoring.

The specimens were positioned within the experimental setup. The distractor tips were placed precisely at a depth of 2 cm, resting upon the posterior segment of the osteotomy line. Force measurements started after an initial distraction of 8 mm. Gradual distraction was achieved by manually rotating the rotation arm at a consistent pace. Simultaneously, the osteotomy line opening was monitored using a digital caliper with an accuracy of one‐tenth of 1 mm. The required force for distraction (RFD) was measured using the force gauge linked to the load cell at 0.5 mm intervals. Distraction continued until the maximum allowable limit, defined by the length of the rail system, was reached. Distraction forces were measured for each 0.5 mm opening in the range of 8–15 mm for all cadavers (see Figure 2).

Statistical analysis was performed using the R 3.3.0 programme (Bell Labs). Given the absence of normally distributed data, nonparametric tests were applied. The Mann–Whitney *U* test was employed to compare RFD values between groups, and the Pearson test was used to assess the correlations between distraction and RFD in both groups. The level of significance was set at *p* = 0.05.

## RESULTS

In the ILC group, the average age of the cadavers was 82.1 (±8.2) years, the average body mass index (BMI) was 21 (±3.4) kg/m², the average height was 1.62 mm and the average weight was 56.1 (±13.1) kg. In the FLC group, the average age of the cadavers was 82.8 (±5.6) years, the average BMI was 20 (±2) kg/m^2^, the average height was 1.6 m and the average weight was 52 (±7.9) kg (Table 1).

**Table 1:** Characteristics of cadavers.

Characteristics	ILC group	FLC group	*p* Value
Age (years)	79 (69–97)	85 (75–89)	n.s
Height (m)	1.60 (1.60–1.85)	1.60 (1.59–1.62)	n.s
Weight (kg)	56 (44–83)	49 (44–63)	n.s
BMI (kg/m²)	22.1 (17.4–28.3)	20.1 (17.4–22.1)	n.s
Gender			
Male	1 (20%)	1 (20%)	n.s
Female	4 (80%)	4 (80%)	

Abbreviations: BMI, body mass index; FLC, fractured lateral cortex; ILC, intact lateral cortex; n.s: nonsignificant.

A statistically significant difference in RFD between the two groups was evident for each 0.5 mm distraction applied, consistently favouring the ILC group. The RFD values recorded in the FLC group were 8.8%–13.2% of those observed in the ILC group. In simple terms, the RFD values in FLC group were one‐seventh to one‐twelfth of the values registered for the equivalent millimetre of distraction in the ILC group (Table 2).

**Table 2:** Comparison of median force required for distraction.

Amount of distraction (mm)	ILC group RFD	FLC group RFD	*p* Value
	Median (range) (N)	Median (range) (N)	
8	15.9 (10.9–21.3)	2.1 (0.8–7.0)	<0.05
8.5	21.4 (14.1–24.8)	2.8 (1.0–8.3)	<0.05
9	26.7 (16.6–33.8)	3.4 (1.2–6.5)	<0.05
9.5	35.6 (18.5–40.4)	3.9 (1.4–7.3)	<0.05
10	38.3 (19.1–58.0)	4.5 (1.5–5.5)	<0.05
10.5	37.6 (21.2–59.5)	4.7 (1.8–6.8)	<0.05
11	42.7 (22.5–60.3)	5.1 (2.0–7.4)	<0.05
11.5	49.1 (23.8–65.1)	5.2 (2.3–8.2)	<0.05
12	57.0 (25.3–58.9)	5.3 (2.5– 8.7)	<0.05
12.5	59.3 (26.9–65.6)	5.4 (2.7–10.0)	<0.05
13	56.5 (27.7–56.5)	5.0 (3.2–9.6)	<0.05
13.5	56.3 (27.0–73.9)	5.6 (4.2–10.0)	<0.05
14	57.8 (26.6–78.2)	5.5 (5.0–14.0)	<0.05
14.5	58.9 (25.3–80.2)	5.9 (5.0–11.1)	<0.05
15	58.3 (24.7–84.5)	6.5 (5.4–14.0)	<0.05

*Note*: *p* < 0.05 statistically significant.

Abbreviations: FLC, fractured lateral cortex; ILC, intact lateral cortex; N, Newton; RFD, required force for distraction.

A significant difference emerged between the groups when comparing the median RFD values within the 8–12 and 8–15 mm ranges. A significant difference emerged between the groups for both ranges (*p* < 0.05). In the ILC group, the initial (8 mm) median RFD was recorded at 15.9 N, whereas in the FLC group, this value was recorded at 2.1 N. Notably, there was an approximate eightfold difference in baseline RFD between the two groups. The fracture of the lateral cortex resulted in an 86.7% RFD reduction compared to the baseline value (*p* < 0.05).

In the ILC group, RFD started at a median of 15.9 N at 8 mm of distraction and exhibited a relatively linear progression, increasing up to 12.5 mm. Notably, at 10 mm of distraction, RFD values reached 38.3 N (approximately 2.4 times the initial value). At 12.5 mm, distraction surged remarkably to 59.3 N (approximately 3.7 times the initial value). In essence, within the ILC group, the most substantial increase in RFD was observed in the 8–10 mm distraction range, whereas the distraction within the 10–12.5 mm distraction range increased in more moderate increments. Beyond 12.5 mm of distraction in the ILC group, RFD remained relatively linear and plateaued. At 15 mm of distraction, the RFD was 58.3 N (again, approximating 3.7 times the initial value). Ultimately, in the ILC group, the progressive distraction led to an increase in RFD up to 12.5 mm, beyond which the RFD stabilised without further enhancement and plateaued.

**Figure 3:**
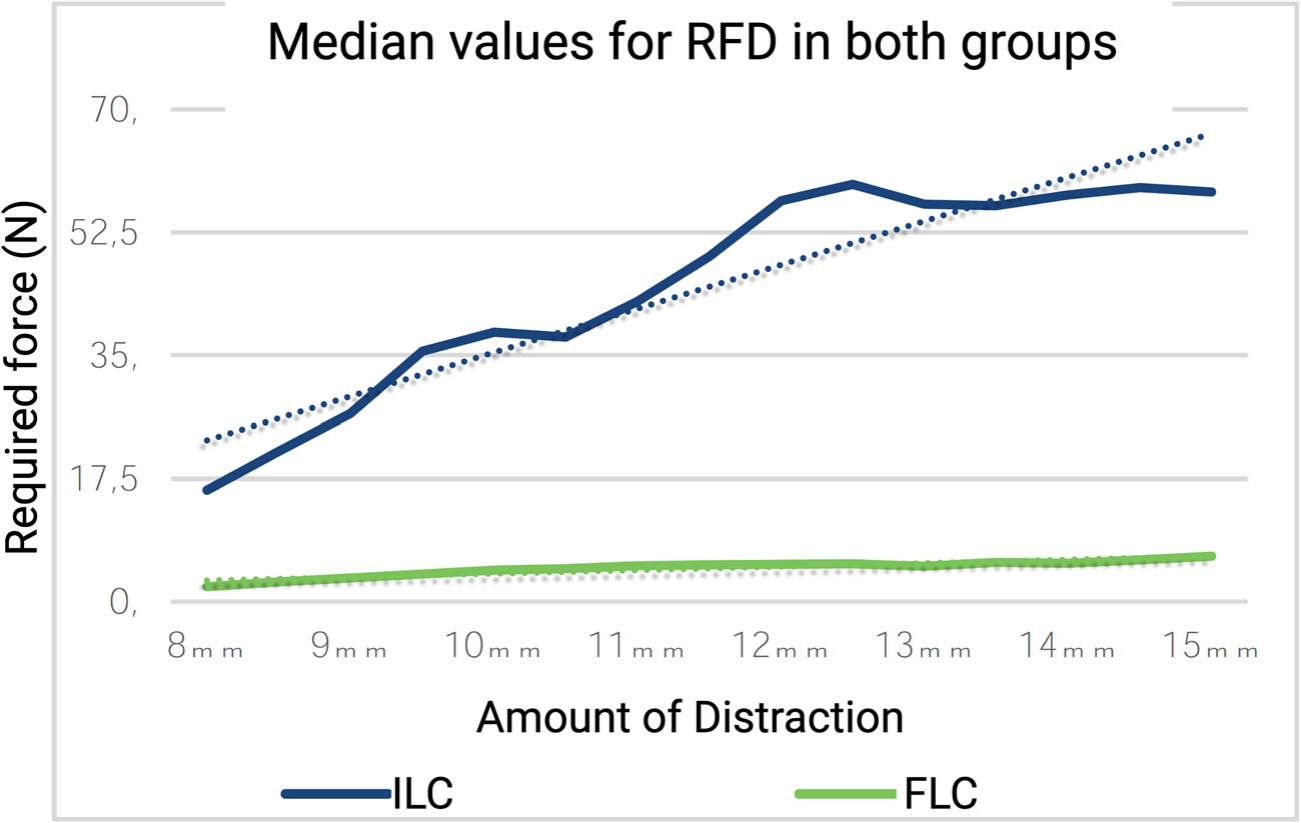
Median values for both groups regarding amount of distraction and required force. FLC, fractured lateral cortex; ILC, intact lateral cortex; RFD, required force for distraction.

In the FLC group, the RFD started at a median of 2.1 N at 8 mm of distraction. It progressively increased to 4.5 N at 10 mm of distraction and further to 5.4 N at 12.5 mm of distraction. The values showed an increase of approximately 2.1 times the initial RFD measurement at 10 mm and roughly 2.6 times the initial measurement at 12.5 mm. As depicted in Figure 3, although the RFD values in the FLC group were initially lower, they followed a relatively linear trend and consistently remained lower than those in the ILC group throughout the distraction period (see Figure 3). Nevertheless, the RFD increase became more limited as distraction increased. Beyond 12.5 mm of distraction, the RFD changes in the FLC group exhibited greater variability, as in the ILC group, although the deviation from the linear trend was comparatively less pronounced. At 15 mm of distraction, the RFD value was 6.5 N (approximately three times the initial value of 2.1 N). In essence, throughout the distraction, the RFD values in the FLC group remained proportionally lower than those in the ILC group.

## DISCUSSION

Our study provides compelling evidence that LHFs lead to substantial reductions in the force required for distraction in MOWHTO. Notably, in the FLC group, we observed an RFD decrease exceeding 90% compared to the ILC group. Although studies have indirectly inferred this phenomenon, our study is the first to demonstrate it with biomechanical evidence. We have shown that medial distraction LHFs during MOWHTO lead to reduced forces applied along the osteotomy line. Hence, as the osteotomy line is progressively distracted during surgery, a sharp decline in the force required for distraction could indicate an LHF, providing a warning for additional lateral column fixation.

In the ILC group, we observed a relatively linear increase in RFD up to 12.5 mm of distraction. However, beyond this point, the median RFD value plateaued. This behaviour suggests that the 1 cm of lateral cortex preserved in compliance with the osteotomy technique permitted elastic deformation up to 12.5 mm, at which point plastic deformation began. A previous study by Agneskirscher et al. underscored the importance of conducting distraction ‘gently’. The authors highlighted the need to slow down the process as the degree of opening increases, while maintaining elastic deformation, to enable the bone to adapt to its new form [1]. However, a recent study [4] conducted on synthetic bone models challenged these conclusions and reported no differences in LHFs related to distraction speed. Another study [5] reported increased lateral cortex fracture rates in cases where the openings exceeded 12.5 mm. Our results implicitly support the latter findings, as distractions surpassing 12.5 mm were associated with a greater susceptibility to FLCs.

In our study, we found that the forces required for distraction were higher in the ILC group than those in the FLC group. In the ILC group, the forces increased across all samples up to 12.5 mm, after which they varied between the samples. We deduced that the phase marked by an increase in force until it plateaued signified the elastic deformation phase, while the subsequent plateau corresponded to the plastic deformation phase [17]. It is essential to acknowledge that the fate of the lateral cortex may vary according to an individual's unique bone structure. This was exemplified by the diverse force requirements across all samples for the same degree of distraction.

While different variables, such as hinge positioning, osteotomy width, age and BMI, can be risk factors for LHF, the ultimate risk factor is the amount of distraction [8, 9, 13]. However, it is worth noting that LHFs can occur even with distractions below the commonly reported critical limit of 12.5 mm. This suggests the need for a case‐specific strategy to prevent LHFs. To implement such a personalised approach, future clinical evaluations of forces applied during surgical medial distraction procedures could be valuable. Türkmen et al. [24] conducted a comparative analysis of uniplanar and biplanar osteotomy techniques, focussing on the occurrence of LHFs. Their findings, based on a synthetic bone model, revealed that LHFs occurred at a lower amount of distraction in monoplanar osteotomy, and the force applied just prior to the fracture was notably lower in the uniplanar osteotomy. However, some studies in clinical settings have challenged these findings, showing a lower incidence of LHFs in uniplanar osteotomies [19]. The testing device employed by Türkmen et al. [24] resembles the one used in our research. LHFs were analysed together with distraction forces in this study; however, the results for the temporal course of applied forces reported in [24], which followed a parabolic pattern throughout the distractions, did not match the results of our study. While the differences may have stemmed from the use of different models, we assert that our study more accurately replicates the clinical scenario.

Our study has several limitations. Considering that our study was a cadaveric study, its capacity to accurately simulate clinical conditions was restricted. The advanced age of the cadavers used (an average age of 82 years) might have affected joint status and bone quality, potentially leading to forces lower than those observed in more typical clinical circumstances. Given that high tibial osteotomy is rarely performed beyond the age of 65 years, this disparity might have contributed to an earlier‐than‐anticipated occurrence of FLCs. Additionally, the manual operation of the distraction device, leading to potentially inconsistent speed and torque, might have caused momentary RFD variations. Variations may have resulted from possible minor but continuously changing fluctuations in the measured force, as observed by an examiner. Different thresholds for the critical limit for distraction can be defined in different settings since the 12.5 mm critical threshold may vary with the age of the patient, hinge size and osteotomy technique (uniplanar vs. biplanar). Despite these limitations, our study stands as an original study in which we characterised distraction forces during medial open wedge high tibial osteotomy on human cadaveric specimens and evaluated the influences of LHF on RFDs.

## CONCLUSION

LHFs during MOWHTO are associated with a significant reduction in the force required for distraction. However, a sudden decrease in distraction force during distraction may indicate an LHF. Possible applications of force measurement devices for use during distraction could provide surgeons with valuable insights and immediate intraoperative warnings for LHFs.

## AUTHOR CONTRIBUTIONS

Arman Vahabi and Baran Soykan wrote the paper. Baran Soykan, Semih Aydoğdu and Bora Uzun performed experiments. Bora Uzun, Baran Soykan, Elcil Kaya Biçer and Semih Aydoğdu analysed the data. Bora Uzun, Semih Aydoğdu and Elcil Kaya Biçer reviewed the paper.

## CONFLICT OF INTEREST STATEMENT

The authors declare no conflict of interest.

## ETHICS STATEMENT

Ethical approval was obtained from the Ege University Faculty of Medicine Ethics Committee (Dated 17/03/2016, Num:16‐1.1/44).

## Data Availability

Data set for that study is available from corresponding author upon request.
